# Food Products as Sources of Protein and Amino Acids—The Case of Poland

**DOI:** 10.3390/nu10121977

**Published:** 2018-12-13

**Authors:** Hanna Górska-Warsewicz, Wacław Laskowski, Olena Kulykovets, Anna Kudlińska-Chylak, Maksymilian Czeczotko, Krystyna Rejman

**Affiliations:** Department of Organization and Consumption Economics, Faculty of Human Nutrition and Consumer Sciences, Warsaw University of Life Sciences, 02-787 Warsaw, Poland; waclaw_laskowski@sggw.pl (W.L.); olena_kulykovets@sggw.pl (O.K.); anna_kudlinska_chylak@sggw.pl (A.K.-C.); maksymilian_czeczotko@sggw.pl (M.C.); krystyna_rejman@sggw.pl (K.R.)

**Keywords:** protein, amino acids food sources, protein food sources

## Abstract

The aim of this study was to identify the food sources of protein and 18 amino acids (AAs) in the average Polish diet. The analysis was conducted based on the 2016 Household Budget Survey (HBS) on the consumption of food products from a representative sample of 38,886 households (*n* = 99,230). This survey was organized, conducted and controlled by the Central Statistical Office, Social Surveys and Living Conditions Statistics Department in cooperation with the Statistic Office in Łódź based on the recording of expenditures, quantitative consumption, and revenues in budget books for one month. 91 food products from 13 food categories (e.g., meat and meat products, grain products) consisting of 42 food groups (e.g., red meat, milk, cheese) were analyzed to determine protein and amino acid intake from these products. Three categories delivered 80.9% of total protein (meat and meat products: 38.9%; grain products: 23.9%; and milk and dairy products: 18.1%). The branched-chain amino acids (BCAAs: leucine, isoleucine and valine) were delivered mainly by meat and meat products (39.9%; 41.3% and 37.4%, respectively). Meat and meat products were also the most important source for other essential amino acids (EAAs: lysine 49.2%, histidine 46.6%, threonine 44.7%, tryptophan 41.4%, phenylalanine 35.3%, and methionine 44.2%). In terms of the contribution of the non-essential or conditionally essential amino acids to the average Polish diet, most important were grain products (for cysteine: 41.2%; glutamic acid: 33.8%; proline: 34.1%), and meat and meat products (for tyrosine: 38.3%; arginine: 46.1%; alanine: 48.7%; aspartic acid: 41.7%; glycine: 52.5%; serine: 33.6%). Five clusters were identified to assess the impact of socio-demographic and economic factors on the protein supply. The largest impact was observed for respondent education, degree of urbanization, study month, and usage of agricultural land. The shares of animal food in total protein supply amounted to 66.5% in total population and varied from 56.4% to 73.6% in different clusters.

## 1. Introduction

Protein is a very important dietary macronutrient required for life [1,2,3,4,5,6] with various metabolic and physiologic functions, including the regulation of appetite, food intake, body weight, and body composition [7,8]. Its role in the regulation of blood pressure, glucose and lipid metabolism, bone metabolism, and the immune system [9,10] is also described in the scientific literature [9].

Taking into consideration the role of proteins and healthy eating patterns related to protein, it is important to analyze the consumption of food with respect to the share of particular food groups in the contribution of energy and nutrients. As far as protein and amino acids are concerned, type of protein to be eaten, protein quality and protein density should be analyzed [1]. The requirements for dietary protein are aimed at providing the minima for nine essential amino acids (EAAs) [11,12] or indispensable amino acids (IAAs) [13]. These are necessary for new protein structures and cannot be produced by the human body in physiologically significant amounts, and therefore must be supplied as crucial components of a balanced diet [11,12,13]. The EAAs include histidine, isoleucine, leucine, valine, lysine, threonine, phenylalanine, methionine, and tryptophan. Three of the nine EAAs (leucine, valine, and isoleucine) are the branched-chain amino acids (BCAAs) [12]. Some researchers have analyzed the role of BCAAs in muscle protein synthesis [7,11,12,14,15,16,17,18,19,20,21]. To the other amino acids belong 11 conditionally essential amino acids (CEAAs) and non-essential amino acids (NEAAs) [12,13].

There are a variety of proteins from a wide spectrum of food sources such as meat, milk, egg, soy, and other plants. Animal foods, in contrast to plant food, are perceived to have higher protein to energy ratios and better digestibility of protein and amino acids [6,7,9,22,23,24,25]. A US population study indicated that the share of animal food (including dairy products) in the total protein consumption amounted to 62% [26]. Fish, poultry, and red meat were the main sources of protein [7]. At the same time, meat and meat products contributed more to zinc, vitamin B12, phosphorus and iron intakes than plant food; however, plant products are higher contributors of fiber, vitamin E and magnesium [27]. Red meat—an important part of the human diet—provides high biological value protein and essential nutrients, some of which are more bioavailable than in alternative food sources [8]. Protein supply is a concern when the intake of both meat and dairy products is reduced [14]. At the same time, consuming a variety of protein food sources (meat, dairy products, fish, cereals, legumes) is advocated to ensure adequate nutrient intakes, as recommended by national guidelines [28].

These aspects are analyzed in economically developed countries [29] and in Western countries due to the development of various new dietary protein patterns in recent years, such as vegan, vegetarian and flexitarian diets [30]. The literature describes level of protein intake [26,31,32], dietary protein patterns in general and in relation to plant, animal and composite protein subgroups [33], and novel protein sources in terms of their possible protein delivery in the future [34].

The main aim of this study was to identify the food sources of protein and 18 amino acids in the average Polish diet, with special attention given to the rankings of food categories and groups in the contribution of protein and amino acids. It is the first attempt to estimate the sources of amino acids in the average Polish diet. Such information can be useful for dietary and health specialists to assess the quality of diets and to meet nutrient recommendations.

## 2. Methods

### 2.1. Study Overview

Only food sources of protein and 18 amino acids were analyzed. The 18 amino acids examined in this study were: histidine (His), leucine (Leu), isoleucine (Ile), lysine (Lys), valine (Val), threonine (Thr), tryptophan (Trp), phenylalanine (Phe), methionine (Met), cysteine (Cys), tyrosine (Tyr), arginine (Arg), alanine (Ala), aspartic acid (Asp), glutamic acid (Glu), glycine (Gly), proline (Pro) and serine (Ser).

### 2.2. Sample Selection

HBS is a representative method organized, conducted and controlled by the Central Statistical Office, Social Surveys and Living Conditions Statistics Department in cooperation with the Statistic Office in Łódź. The representative sample of the 2016 HBS consisted of 38,886 households (*n* = 99,230) which participated in the survey [35] by recording expenditures, quantitative consumption, and revenues in special budget books for one month. To select a representative sample, a two-stage, layered scheme was used. The sampling units of the first stage were area survey points and in the second one, flats and apartments were selected. The basis for the sampling frame for 1st degree units was the list of statistical regions developed for the needs of the National Census, updated each year with changes resulting from the administrative division of the country. The second-degree selection was made up of lists of inhabited flats and apartments in randomly selected area survey points, developed by statistical offices. In 2016, 911 area points were located in cities and 665 in rural areas [35,36]. Detailed information related to sample selection was presented in our previous paper [37]. The data on each household participating in the survey are from the “Budget Diary” and “Household’s Statistical Sheet”. Each household kept records of expenditures, quantitative food consumption and revenues for one month [35,36].

In the 2016 HBS sample population, the share of women was 52.4%. In the age structure, the largest share was represented by the following groups: 25–34 years (12.6%), 35–44 years (13.8%), 45–54 years (12.5%), 55–64 years (15.8%), 65 years and over (17.1%). The share of people under 25 and children was 28.1% in total. The sample included four main types of households: employees (*N* = 17,877 households, *n* = 55,799 people), farmers (*N* = 1689, *n* = 6481), self-employed (*N* = 2500, *n* = 7970), retirees and pensioners (*N* = 13,323, *n* = 25,195). The structure of households by the number of people was as follows: one-person (*N* = 7590, *n* = 7590), two-person (*N* = 12,085, *n* = 24,170), three-person (*N* = 7300, *n* = 21,900), four-person (*N* = 6130, *n* = 24,520), five-person (*N* = 2363, *n* = 11,815), six and more-person (*N* = 1418, *n* = 9235). In terms of educational level, the largest groups in the sample population were characterized by basic vocational (27.4%), upper secondary vocational (19.8%), higher (18.9%), and primary (16.4%) education [35].

Data on the consumption of food products were converted into one person per month using the information on the number of persons in the household and the number of days of using home nutrition. Such converted data on consumption should be regarded as a comprehensive diet [38].

### 2.3. Food Grouping

The HBS analyzed 91 food products. For the purposes of analysis of protein and amino acid contributions, there were 42 food and beverage groups in 13 food and beverage categories (Table 1). The food classification scheme was adapted from one published earlier [31,39,40,41]. The food classification included foods commonly consumed by Polish consumers [35].

### 2.4. Statistical Analysis and Data Presentation

To calculate the protein and amino acid content of the food, the ‘Nutritive Value Tables for Foods and Meals’ (4th ed., 2017) [42] were used. This edition was developed and updated by the Food and Nutrition Institute with special attention given to new products and technological modifications taking place in the Polish food industry. In comparison to the previous edition (3rd ed., 2005), the content of fatty acids and amino acids was taken into account; therefore, calculations required for the purposes of this study were possible. From the base of 1100 products and assortment items, 930 products were selected. The weights of the known or estimated proportions of the consumption of given products relative to others in the group were applied, if necessary, to calculate the average protein and amino acids contents. This database was inputted into the R program (v 3.0.2), a system and environment for statistical computation [43,44,45], and used to calculate values and contents of protein and amino acids in food consumed in each of the 38,886 households (*n* = 99,230). A weight of corrections was used to analyze the diversity of household structure and to improve the representativeness of the results [38,46,47,48]. Means and standard errors of energy were calculated for 13 food and beverage categories and 42 food and beverage groups. Means of the protein and amino acid intakes were expressed as percentages of the total dietary intake of the analyzed nutrient. Percentages of protein and amino acids supply of food categories and groups are presented in rank order.

To assess the impact of socio-demographic and economic factors on the protein supply in the average Polish diet, exploratory data analysis (EDA) was applied. In the literature, the EDA is described as a method or approach to gaining new insights into data, identifying important factors in the data and understanding relationships [47,49,50]. We used a cluster analysis as an exploratory tool to sort data into groups, which is widely discussed in the scientific literature [51,52,53,54,55,56,57,58,59]. In our calculations, eight food categories of protein sources were considered, which statistically account for the delivery of 98.7% of the total protein. Based on a multidimensional exploratory analysis of the percentage share of protein from theses eight sources, five clusters (groups) were identified. For this purpose, the Neural Networks module available in the Statistica 13.3 program was used and Kohonen Neural Network was selected from the list of available networks [60]. The division into five clusters is characterized by an averaged correlation measure (correlation ratio) of almost 0.5.

The description of clusters includes the following socio-demographic and economic features: respondent education, degree of urbanization, study month, usage of agricultural land, socio-economic type of household (i.e., households of employees, farmers, self-employed, living on unearned sources, and retirees and pensioners), size of the village, family life phase, age, income measured according to the quintiles group, province, assessment of the household’s financial situation, number of people in the household, region and sex. For each such feature, and considering the exploratory classification obtained, a correlation table was created together with a chi2 test and a measure of Cramer’s correlation. The most important data related to the share of main food categories in terms of the contribution of protein and 18 amino acids are presented in Section 3 “Results”.

Detailed data related to the share of 13 food and beverage categories and 42 food and beverage groups in terms of the contribution of total protein and all analyzed AAs are shown in the Supplemental Section.

## 3. Results

Sources of protein and 18 amino acids from the main food and beverage categories are shown in Table 2, Table 3, Table 4, Table 5 and Table 6, which appear in this article, and detailed data are presented in Tables S1–S19 in the Supplemental Section.

### 3.1. Protein Sources

Sources of protein calculated for food categories are shown in Table 2, and for food groups in Table S1 (Supplemental Section). The three highest sources of protein were meat and meat products (39.0%), grain products (23.9%), and milk and dairy products (18.1%) (Table 2). The highest ranked food group sources of protein were meat products (17.4%), bread, rolls, bread products (16.5%), red meat (9.9%), poultry (9.7%), and cheese (5.6%) (Table S1).

### 3.2. Food Sources of BCAAs

The main food sources of BCAAs calculated in food categories are shown in Table 3. The detailed data related to food categories and groups are presented in Supplemental Tables S2–S4.

The main food categories that are contributors to leucine consumption were meat and meat products (39.9%), grain products (22.1%), and milk and dairy products (20.0%) (Table 3). When considering food groups, the main sources of leucine were processed red meat products (18.4%), bread, rolls and bread products (14.9%), red meat (10.5%), poultry (9.0%), and milk (6.2%) (Table S2).

Meat and meat products contributed 41.3% of isoleucine in the average Polish diet. The other sources of isoleucine were grain products (21.3%), and milk and dairy products (19.1%) (Table 3). When considering food groups, the main sources of isoleucine were processed meat products (19.2%), bread, rolls and bread products (14.8%), red meat (10.3%), poultry (10.1%), and pork (6.3%) (Table S3).

The top contributors of valine were: meat and meat products, grain products, and milk and dairy products, delivering 80.3% of total valine supply (Table 3) and as detailed food groups: meat products (16.6%), bread, rolls and bread products (15.3%), poultry (9.5%), red meat (9.5%), and milk (6.5%) (Table S4).

### 3.3. Food Sources of Other EAAs

The main food sources of other EAAs (lysine, histidine, threonine, tryptophan, phenylalanine, and methionine) from food categories are shown in Table 4. Detailed data related to all food categories and groups are presented in Supplemental Tables S5–S10.

Meats and meat products are a very important source of lysine, delivering almost half of total intake (Table 4). The two other main food categories which were contributors of lysine were milk and dairy products (21.5%), and grain products (11.0%). The highest ranked food group sources of lysine were meat products (22.3%), poultry (12.4%), red meat (12.3%), bread, rolls and bread products (7.5%), and milk (7.4%) (Table S5).

Meat and meat products were the sources for c.a. 46.5% of histidine in the average Polish diet (Table 4). The other two largest sources of histidine were grain products (19.2%), and milk and dairy products (17.0%). The highest-ranked food group sources of histidine were processed meat products (21.2%), bread, rolls and bread products (13.1%), red meat (11.8%), poultry (11.6%), and cheese (5.8%) (Table S6).

Meats and meat products were the source of nearly 45% of threonine in the average Polish diet (Table 4). The other largest sources of threonine were grain products (18.5%), and milk and dairy products (17.6%). When considering food groups, the main sources of threonine were meat products (20.6%), bread, rolls and bread products (12.7%), red meat (12.3%), poultry (9.8%) and vegetables excluding potatoes (5.3%) (Table S7).

The level of tryptophan derived from food is presented in Table 4 and Table S8. The main food category contributors of tryptophan in the average Polish diet were meat and meat products (41.4%), grain products (19.2%), and milk and dairy products (18.9%) (Table 4). When considering food groups, the top five ranked foods were meat products (18.4%), bread, rolls and bread products (12.9%), red meat (10.6%), poultry (10.6%), and cheese (6.2%) (Table S8).

Meats and meat products were the sources for c.a. 35% of phenylalanine in the average Polish diet (Table 4). Phenylalanine was also delivered by grain products and milk and dairy products. Detailed data on food sources of phenylalanine are presented in Table S9 with main rank positions related to: bread, rolls and bread products (18.0%), meat products (16.4%), red meat (9.2%), poultry (7.9%), and cheese (6.2%).

Meats and meat products contributed 44.2% of methionine (Table 4) in the average Polish diet. The next two main food categories—grain products and milk and dairy products—contributed nearly 39% of methionine. Detailed data showed the highest ranked food sources of methionine to be meat products (19.9%), bread, rolls and bread products (13.7%), poultry (11.4%), red meat (11.1%), and cheese (5.6%) (Table S10).

### 3.4. Food Sources of CEAAs

The main food sources of CEAAs calculated in food categories are shown in Table 5. Detailed data related to all food categories and food groups are presented in Supplemental Tables S11–S15.

Grain products are very important sources of cysteine, delivering 41.7% of total intake (Table 5). The other main food category as a contributor to cysteine was meat and meat products (31.4%). The highest-ranked food group sources of cysteine were bread, rolls and bread products (29.0%), meat products (14.6%), red meat (8.1%), poultry (7.1%), and flour, bran and cooking ingredients (6.3%) (Table S11).

The three highest sources of tyrosine were meat and meat products (38.3%), milk and dairy products (23.6%), and grain products (20.6%) (Table 5). When considering food groups, the main sources of tyrosine were meat products (17.7%), bread, rolls and bread products (14.5%), red meat (10.0%), poultry (8.8%), and cheese (8.3%) (Table S12).

Meat and meat products were the sources for c.a. 46.1% of arginine in the average Polish diet (Table 5). The other largest sources of arginine were meat products (21.0%), and poultry (11.3%). When considering food groups, the main sources of arginine were meat products (20.9%), bread, rolls and bread products (14.3%), red meat (12.1%), poultry (10.9%), and vegetables excluding potatoes (7.2%) (Table S13).

Meat and meat products are very important sources of glycine, delivering about half of the total intake (Table 5). The two other main food categories that were contributors of glycine were grain products (21.05%) and milk and dairy products (7.84%). The highest-ranked food group sources of glycine were meat products (21.0%), poultry (15.4%), bread, rolls and bread products (14.4%), red meat (12.8%), and vegetables excluding potatoes (5.4%) (Table S14).

For proline, the grain products were the main contributors, delivering 34.1% of total supply (Table 5). The next positions were occupied by meat and meat products (26.7%) and milk and dairy products (24.8%). When detailed data are taken into consideration, the share of four food groups (bread, rolls, and bread rolls; meat products; cheese; and poultry) exceeded 50% of the total supply (Table S15).

### 3.5. Food Sources of NEAAs

The main food sources of NEAAs calculated in food categories are shown in Table 6. The detailed data related to food categories and food groups are presented in Supplemental Tables S16–S19.

Aspartic acid was delivered to the average Polish diet by the following food categories: meat and meat products (41.7%), grain products (16.7%), and milk and dairy products (14.3%) (Table 6) and detailed food groups: meat products (18.5%), bread, rolls and bread products (11.4%), poultry (10.7%), red meat (10.5%) and vegetables without potatoes (7.1%) (Table S16).

The main contributors of glutamic acid to the average Polish diet were grain products delivering c.a. 1/3 of total supply (Table 6). The other important food categories in glutamic acid included meat and meat products (30.9%) and milk and dairy products (19.0%). In food group specifications, the highest ranks were obtained by: bread, rolls and bread products (23.2%), meat products (14.0%), red meat (7.8%), poultry (7.7%) and cheese (6.2%) (Table S17).

Meat and meat products delivered 33.6% of serine (Table 6). The other main food categories contributing serine were: grain products (24.0%), and milk and dairy products (21.1%). The detailed specification of serine contribution indicated the following food groups: bread, rolls and bread products (16.9%), meat products (15.1%), red meat (8.4%), poultry (8.3%), and cheese (6.5%) (Table S18).

The top contributors of alanine were the following main food categories: meat and meat products (48.7%), grain products (19.2%), and milk and dairy products (11.9%) (Table 5) and food groups: meat products (21.2%), bread, rolls and bread products (13.1%), poultry (12.9%), red meat (12.1%), and vegetables excluding potatoes (5.6%) (Table S19).

### 3.6. Summary of AAs Sources

Meat and meat products, grain products, and milk and dairy products reviewed jointly were important sources of amino acids delivering more than 80% of proline, glutamic acid, tyrosine, histidine, methionine, cysteine, lysine, glycine, leucine, isoleucine, phenylalanine, threonine, and valine (Table 7). In the case of glycine, lysine, alanine, histidine, arginine, threonine, methionine, aspartic acid, tryptophan and isoleucine, meat and meat products were responsible for more than 40% of daily intake. Grain products delivered more than 30% of the average daily intake in the case of cysteine, glutamic acid, and proline. An important contribution (more than 20% of daily intake) of milk and dairy products to the daily consumption of proline, tyrosine, serine, valine, lysine, and leucine was observed.

### 3.7. Cluster Analysis Based on Protein Sources

**Figure 1:**
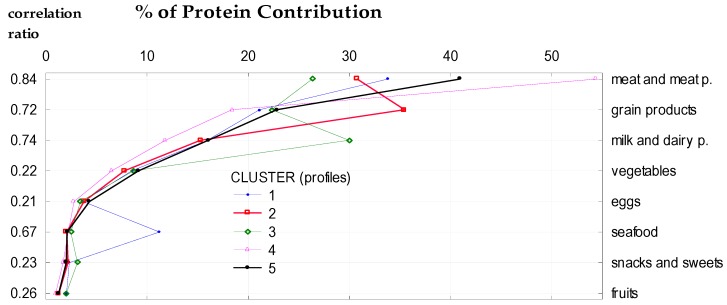
Cluster analysis of population sample in relation to the food categories contribution to total protein supply. p.—products.

To assess the impact of socio-demographic and economic factors on the protein supply in the average Polish diet, eight categories of protein sources as classification features were considered (Table 8). Five clusters were identified, which ensured the highest value of correlation ratio (0.48). The largest impact on the total protein intake in clusters was observed for the following factors: respondent education, degree of urbanization, study month, and usage of agricultural land (Table 9). In individual clusters, a different value of animal vs. plant protein ratio and various shares of eight food categories in total protein intake were observed. The shares of animal food in total protein supply amounted to 66.5% in total population and varied from 56.4% in cluster 2 to 73.6% in cluster 4. In comparison, plant food was the source of less protein (33.5% in total population, 26.4% in cluster 4, and 43.6% in cluster 2), (Table 10). Considering the eight categories as protein sources, the largest differences among clusters were found for meat and its products, which were greater than those for cereal products, and milk and dairy products. However, it is worth noting that one cluster differed from the others primarily by the percentage of seafood protein (Figure 1).

## 4. Discussion

This analysis determined the sources of protein and 18 amino acids in the average Polish diet based on the 2016 HBS. It also ranked 13 food and beverage categories and 42 food and beverage groups including 91 food products. The main contributors of protein were meat and meat products (39.0% of total protein supply), grain products (23.9%), and milk and dairy products (18.1%). The combined share of these food categories exceeded 80% of the total protein supply. In the category of meat and meat products, the largest contributors of protein were meat products (processed red and poultry meat products) 17.4%, red meat (beef, pork, sheep and goat) 9.9%, and poultry (mainly chicken) 9.9%. In the grain product category, the highest ranked food group in protein contribution was bread, rolls and bread products, delivering 16.5% of total protein supply. In dairy products, three food groups were ranked as important protein sources: cheese (5.6%), milk (5.4%), and yogurts and milk drinks (3.7%).

Three other studies were selected for comparison of food sources of total protein contribution to the average Polish diet: 2003–2006 NHANES (National Health and Nutrition Examination Survey 2003–2006) [31], 2007–2010 NHANES (National Health and Nutrition Examination Survey 2007–2010) [26], and 2011–2012 NNPAS (Australian National Nutrition and Physical Activity Survey 2011–2012) [32]. The 2003–2006 NHANES research identified the main sources of protein as follows: poultry (14.4%), beef (14%), pork, ham, bacon (5.7%), fish and shellfish (5.0%), and frankfurters, sausages, and luncheon meats (4.4%) [31]. In the average Australian diet, the contribution of meats to protein supply amounted to 49% (2011–2012 NNPAS). Within the red meat category, beef was the most popular meat type, followed by lamb and pork. In the poultry category, chicken was the major meat type, together with other poultry meats such as duck, turkey [32]. To summarize the comparison of protein sources, meat and meat products contributed 39.0% of total protein in the average Polish diet, 46.0% in the American diet and 49.0% in the Australian diet [26,32]. Milk and dairy products delivered 16% and 18.1% of total protein to the American and Polish diets, respectively. The share of milk amounted to 5.5% and 5.4% in the American and Polish diet, while cheese was responsible for 4.3 and 5.6% of total protein supply, respectively [26]. These differences in protein contribution in the Polish, American and Australian diets are related to various dietary patterns determined by consumer preferences, product availabilities and factors determining the food purchasing process.

Our research indicated the impact of 14 socio-demographic and economic factors on the protein intake in the average Polish diet. The largest impact was observed for the following factors: respondent education, degree of urbanization, study month, and usage of agricultural land. We identified five clusters of different animal vs. plant protein ratios and shares of eight food categories in the protein contribution. In the average Polish diet, the share of animal food in total protein supply amounted to 66.5% in the total population and varied from 56.4% to 73.6% among the clusters. In comparison, plant food was the source of less protein (33.5% in total population varying from 26.4% to 43.6%). The 2007–2010 NHANES analysis indicated that the share of animal protein equaled 62%, whereas plant protein represented 30% of total protein intake [26].

There is limited data on the food source of amino acids in diets. Our findings indicated that, in the average Polish diet, BCAAs were delivered by meat and meat products, which contributed 39.8% of leucine, 41.3% of isoleucine, and 37.4% valine. The other main food sources for BCAAs were grain products, and milk and dairy products. The detailed information indicated that processed red and poultry products, and bread, rolls and bread products were the main food group sources in the contribution of BCAAs. These findings should be taken into consideration when considering the quality of vegan, vegetarian and flexitarian diets due to the role of BCAAs in protein synthesis, which is widely described in the scientific literature [8,10,17,61,62,63]. BCAA content is generally higher in animal proteins than plant proteins [8,9], with the highest level in red meat [8].

As far as other EAAs are concerned, in the average Polish diet, meat and meat products were the main food category source of histidine (46.6%), lysine (49.2%), threonine (44.7%), tryptophan (41.2%), phenylalanine (35.3%), and methionine (44.2%). Grain products ranked second in terms of the contribution of other EAAs delivering from 18.4% (for methionine) to 26.3% of phenylalanine. The share of milk and dairy products in the intake of other EAAs amounted to 15–20% of daily intake. It is underlined in the scientific literature that food of animal origin provides important nutrients, including lysine, bioavailable iron and zinc, that are not easily delivered by plant food [64]. There is also discussion in the literature that chicken and turkey are important elements in a balanced diet during growth with specific requirements [65,66].

Considering tryptophan, four food groups delivered more than 50% of average intake in the Polish diet, including meat products (18.4%), bread, rolls, and bread products (12.9%), red meat (10.6%), and poultry (10.6%). This structure of tryptophan contribution to average diets is important due to the role of tryptophan as a precursor of serotonin in food intake and appetite and this is analyzed in the scientific literature [7]. This function of tryptophan is related to carbohydrate-rich, protein-poor meals [67], and eating behavior control, meal size, and body weight [68].

Histidine was delivered to the average Polish diet by meat products (21.2%), bread, rolls, and bread products (13.1%), red meat (11.8%), and poultry (11.6%). Increasing poultry consumption determined the share of poultry in contributing histidine, which is analyzed in the literature [65,66]. For methionine and threonine, the same order of food groups was observed as for histidine. The highest share was noticed in the case of meat products (19.9% for methionine contribution and 20.6% in threonine intake). Subsequent places were occupied by bread, rolls and bread products (13.7% and 12.7%, respectively), poultry (11.4% and 9.8%), and red meat (11.1% and 12.3%). In the case of phenylalanine, in the average Polish diet, bread, rolls and bread products were most important, delivering 18.0% of total intake. The other food groups functioning as phenylalanine contributors were: meat products (16.4%), red meat (9.2%), poultry (7.9%), and cheese (6.2%).

To summarize, it should be stressed that the content of EAAs is analyzed in assessments of diet quality in terms of the capacity of the diet to provide needs for protein synthesis [9,69]. This is especially crucial in the assessment of non-meat diets due to increasing interest in vegetarianism and veganism [10,70,71,72,73,74]. Health professionals should encourage vegetarians to include a variety of protein-rich foods, such as whole grains; legumes; beans, split peas and baked beans; soy products; nuts and seeds [10].

Our findings indicated that CEAAs and NEAAs were mainly delivered by meat and grain products to the average Polish diet. A share of the contribution of meat and meat products exceeding 40% was noted for glycine (52.5%), alanine (48.7%), arginine (46.1%), and aspartic acid (41.7%). For cysteine, tyrosine, glutamic acid and serine, the level of the contribution of meat and meat products amounted to 30–40%; a supply below 30% was identified in the case of proline. Grain products were the main contributors of cysteine, delivering 41.7% of the total intake of this amino acid, glutamic acid (33.8%), and proline (34.1%). The other CEAAs and NEAAs were delivered by grain products at levels of 20–30% (serine, arginine, and tyrosine) and below 20% (aspartic acid, alanine). Milk and dairy products were the largest contributors of CEAAs and NEAAs (above 20%) in the case of proline (24.8%), tyrosine (23.6%), and serine (21.1%). A contribution of this food category at the level of 10–20% of CEAAs and NEAAs was noted for arginine (11.3%), alanine (11.9%), aspartic acid (14.3%), and glutamic acid (18.9%). The lowest share of milk and dairy products (below 10%) was identified for glycine and cysteine.

The 2016 HBS sample range (38,886 households), representative sample selection, consistent approach to classifying food products, use of the HBS methodology to analyze food sources of total protein and 18 amino acids, and animal/plant protein ratios are the strengths of the current study. However, there are some limitations related to the reliance on self-report special budget books, which can under- or overestimate consumption data, even though HBS uses well-established procedures. Additionally, the current edition of ‘Nutritive Value Tables for Foods and Meals’ (4th ed., 2017) includes new products and technological modifications, which may cause difficulties in comparison of current results with data from earlier years. Therefore, further research is needed to identify food sources of other nutrients, and to assess the impact of socio-demographic, and economic factors on the structure of other nutrient contributions to the average Polish diet.

## 5. Conclusions

This population-based study provides a comprehensive analysis of food sources of total protein and 18 amino acids contributing to the average Polish diet. Our findings indicated that the majority of total protein was delivered by three main food categories: meat and meat products, grain products, and milk and dairy products (with combined share exceeding 80% of total protein supply). Concerning the contribution of EAAs to the average Polish diet, the share of meat and meat products ranged from 35.3% for phenylalanine to 49.2% in the case of lysine. Grain products delivered from 10.1% (of lysine) to 26.3% (of phenylalanine) of EAAs, while milk and dairy products contributed 17.0% (for histidine) to 21.5% (of lysine) of EAAs. The share of animal food in total protein supply amounted to 66.5% compared to plant food (33.5%). These results should be taken into consideration in the quality assessment of non-meat diets due to the increasing popularity of vegetarianism and veganism. Knowledge of sources of protein and amino acids can help dietary professionals to develop strategies with a wide spectrum of food products to meet nutrient recommendations for various consumer groups.

## Figures and Tables

**Table 1:** Food grouping for the purpose of the nutrient source analysis.

Food Categories(13)	Food Groups (42)	Food Products (91)
GRAIN PRODUCTS	bread, rolls, bread products	bread and rolls quick breads and bread products
	rice, cooked grains	rice groats and cereal grains
	flour, bran, cooking ingredients	wheat flour other flours
	pizza, pasta, and other flour dishes	pasta, macaroni, noodle pizza and other flour dishes
	ready-to-eat cereal	breakfast cereals
MEAT AND MEAT PRODUCTS	red meat	beef pork sheep, goat veal
	meat products	processed red meat products processed poultry meat products other meat products
	other meat	liver and organ meat minced meat other meat
	Poultry	chicken poultry excluding chicken
MILK AND DAIRY PRODUCTS	Milk	whole milk reduced fat milk condensed and powdered milk
	Cheese	cheeses
	cottage cheese	cottage cheese
	yogurts and milk drinks	yogurt, milk shakes and other dairy drinks
SEAFOOD	Fish	fresh, chilled or frozen fish
	Shellfish	fresh, chilled or frozen shellfish
	processed seafood	dried, smoked and salted seafood other fish and shellfish products
EGGS	Eggs	eggs
FATS AND OILS	Butter	butter
	oils, olive	olive oil other oils
	other fats	margarine and other plant fats other animal fats
	sour cream	cream
FRUITS	Fruits	apples bananas berries citrus fruits frozen fruits fruits products other fruits peaches and nectarines
	dried fruits and nuts	dried fruits and nuts
VEGETABLES	Potatoes	potatoes potatoes products
	vegetables (excluding potatoes)	beetroot cabbage carrots cauliflower cucumber lettuce onions tomatoes frozen vegetables and mushrooms sour cabbage other vegetables and mushrooms vegetable and mushrooms products
SNACKS AND SWEETS	Chocolate	chocolate powdered cacao powdered chocolate
	Desserts	ice-cream
	Snacks	chips
	sweet bakery products	cakes and pies
SUGARSAND SALT	Honey	honey
	jams, syrups, marmalade	jams syrups marmalade
	Sugar	sugar
	sugar substitutes	sugar substitutes
	Salt	salt
BEVERAGES, NONALCOHOLIC	Juices	fruit juices vegetables and mixed juices
	other beverages	other nonalcoholic beverages
	Water	water
COFFEE, TEA	Coffee	coffee
	Tea	tea
ALCOHOLIC BEVERAGES	Wine	grape wine high alcohol wine other wine wine-based beverages
	Beer	beer lager low-alcohol and non-alcohol beer other beer beer-based beverages
	other alcoholic beverages	liquor and cocktails other alcoholic beverages

**Table 2:** Food category sources of protein contribution to the average Polish diet.

Food Categories	Rank	% of Protein Contribution	Cumulative % of Protein Contribution
meat and meat products	1	39.0	39.0
grain products	2	23.9	62.9
milk and dairy products	3	18.1	81.0
vegetables	4	7.9	88.9
eggs	5	3.5	92.4
seafood	6	2.6	95,0
snacks and sweets	7	2.4	97.4
fruits	8	1.3	98.7
fats and oils	9	0.6	99.3
nonalcoholic beverages	10	0.4	99.7
alcoholic beverages	11	0.3	100.0

**Table 3:** Main food category sources of leucine, isoleucine and valine contribution to the average Polish diet.

Food Categories	Rank	% of Contribution	Cumulative % of Contribution
**Leucine**			
meat and meat products	1	39.9	39.9
grain products	2	22.1	62.0
milk and dairy products	3	20.0	82.0
vegetables	4	7.0	89.0
eggs	5	3.9	92.9
**Isoleucine**			
meat and meat products	1	41.3	41.3
grain products	2	21.3	62.6
milk and dairy products	3	19.1	81.7
vegetables	4	6.9	88.6
eggs	5	4.3	92.9
**Valine**			
meat and meat products	1	37.4	37.4
grain products	2	22.1	59.5
milk and dairy products	3	20.8	80.3
vegetables	4	7.8	88.1
eggs	5	4.3	92.4

**Table 4:** Main food category sources of the contribution of lysine, histidine, threonine, tryptophan, phenylalanine, and methionine to the average Polish diet.

Food Categories	Rank	% of Contribution	Cumulative % of Contribution
**Lysine**			
meat and meat products	1	49.2	49.2
milk and dairy products	2	21.5	70.7
grain products	3	11.0	81.7
vegetables	4	7.5	89.2
Eggs	5	3.3	92.5
**Histidine**			
meat and meat products	1	46.6	46.6
grain products	2	19.2	65.8
milk and dairy products	3	17.0	82.8
vegetables	4	6.5	89.3
seafood	5	3.2	92.5
**Threonine**			
meat and meat products	1	44.7	44.7
grain products	2	18.5	63.2
milk and dairy products	3	17.5	80.7
vegetables	4	7.8	88.5
eggs	5	4.0	92.5
**Tryptophan**			
meat and meat products	1	41.4	41.3
grain products	2	19.2	60.6
milk and dairy products	3	18.9	79.5
vegetables	4	9.0	88.5
eggs	5	4.2	92.7
**Phenylalanine**			
meat and meat products	1	35.3	35.3
grain products	2	26.3	61.6
milk and dairy products	3	19.2	80.8
vegetables	4	7.9	88.7
eggs	5	4.4	93.1
**Methionine**			
meat and meat products	1	44.2	44.2
grain products	2	20.0	64.2
milk and dairy products	3	18.4	82.6
vegetables	4	5.1	87.7
eggs	5	4.9	92.6

**Table 5:** Main food category sources of the contribution of cysteine, tyrosine, arginine, proline, and glycine to the average Polish diet.

Food Categories	Rank	% of Contribution	Cumulative % of Contribution
**Cysteine**			
grain products	1	41.7	41.7
meat and meat products	2	31.4	73.1
milk and dairy products	3	8.5	81.6
vegetables	4	6.4	88.0
eggs	5	5.1	93.1
**Tyrosine**			
meat and meat products	1	38.3	38.3
milk and dairy products	2	23.6	61.9
grain products	3	20.6	82.5
vegetables	4	6.3	88.8
eggs	5	4.0	92.8
**Arginine**			
meat and meat products	1	46.1	46.1
grain products	2	21.0	67.1
milk and dairy products	3	11.3	78.4
vegetables	4	9.6	88.0
eggs	5	4.1	92.1
**Glycine**			
meat and meat products	1	52.5	52.5
grain products	2	21.0	73.5
milk and dairy products	3	7.8	81.4
vegetables	4	7.5	88.9
seafood	5	3.4	92.3
**Proline**			
grain products	1	34.1	34.1
meat and meat products	2	26.7	60.8
milk and dairy products	3	24.8	85.6
vegetables	4	5.4	91.0
snacks and sweets	5	2.4	93.4

**Table 6:** Main food category sources of the contribution of aspartic acid, glutamic acid, serine, and alanine to the average Polish diet.

Food Categories	Rank	% of Contribution	Cumulative % of Contribution
**Aspartic Acid**			
meat and meat products	1	41.7	41.7
grain products	2	16.7	58.4
milk and dairy products	3	14.3	72.7
vegetables	4	13.8	86.5
eggs	5	3.5	90.0
**Glutamic Acid**			
grain products	1	33.8	33.8
meat and meat products	2	30.9	64.7
milk and dairy products	3	10.0	83.7
vegetables	4	7.2	90.9
eggs	5	2.4	93.3
**Serine**			
meat and meat products	1	33.6	33.6
grain products	2	24.0	57.6
milk and dairy products	3	21.1	78.7
vegetables	4	7.7	86.4
eggs	5	5.6	92.0
**Alanine**			
meat and meat products	1	48.7	48.7
grain products	2	19.2	67.8
milk and dairy products	3	11.9	79.7
vegetables	4	7.8	87.5
eggs	5	4.3	91.8

**Table 7:** The shares (in %) of three main food categories in the contribution of amino acids to the average Polish diet.

Amino Acids	Total Share of 3 Food Categories	Meat and Meat Products (share in %)	Grain Products (share in %)	Milk and Dairy Products (share in %)
glycine	81.4	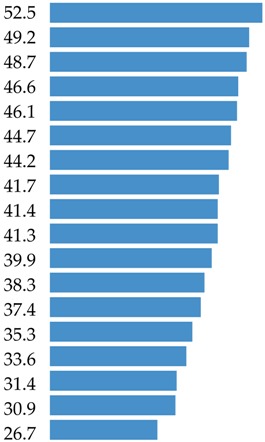	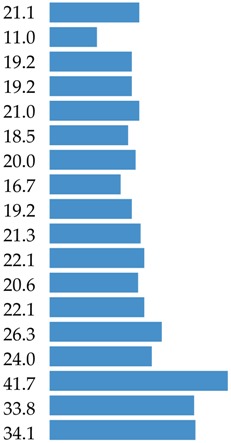	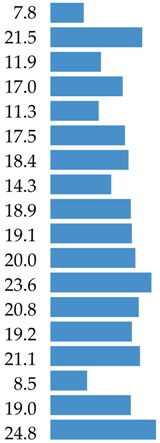
lysine	81.7			
alanine	79.7			
histidine	82.8			
arginine	78.4			
threonine	80.7			
methionine	82.6			
aspartic acid	72.7			
tryptophan	79.5			
isoleucine	81.7			
leucine	82.0			
tyrosine	82.5			
valine	80.3			
phenylalanine	80.8			
serine	78.7			
cysteine	81.6			
glutamic acid	83.7			
proline	85.6			

**Table 8:** Description of cluster analysis: protein sources as classification features.

Food Category	Correlation Ratio
meat and meat products	0.84
grain products	0.72
milk and dairy products	0.74
vegetables	0.22
eggs	0.2
seafood	0.67
snacks and sweets	0.23
fruits	0.26
average	0.48

**Table 9:** Dependence of cluster analysis on socio-demographic and economic factors.

Factors	Cramer Correlation
education	0.158
degree of urbanization	0.138
study month	0.135
usage of agricultural land	0.133
socio-economic type of household	0.118
size of the village	0.114
family life phase	0.105
age	0.101
income (quintile group)	0.100
province	0.099
assessment of financial situation	0.080
number of people in household	0.066
region	0.057
sex	0.033

**Table 10:** Cluster description: animal vs. plant food.

	Sample Population	Cluster 1	Cluster 2	Cluster 3	Cluster 4	Cluster 5
Animal food (in %)	66.5	68.0	56.4	66.5	73.6	66.8
Plant food (in %)	33.5	32.0	43.6	33.5	26.4	33.2
Number of households in cluster (in %)	100	5.7	17.9	19.4	25.5	31.5
Number of people in cluster (in %)	100	5.0	18.3	19.7	24.7	32.3

## Supplementary Materials

The following Tables are available in http://www.mdpi.com/2072-6643/10/12/1977/s1. Supplemental Section: Table S1. Food group sources of protein contribution to the average Polish diet (food categories and groups contributed at least 0.2% of protein), Table S2. Food categories and groups sources of leucine contribution to the average Polish diet (food categories and groups contributed at least 0.2% of leucine), Table S3. Food categories and groups sources of isoleucine contribution to the average Polish diet (food categories and groups contributed at least 0.2% of isoleucine), Table S4. Food categories and groups sources of valine contribution to the average Polish diet (food categories and groups contributed at least 0.2% of valine), Table S5. Food categories and groups sources of lysine contribution to the average Polish diet (food categories and groups contributed at least 0.2% of lysine), Table S6. Food categories and groups sources of histidine contribution to the average Polish diet (food categories and groups contributed at least 0.2% of histidine), Table S7. Food categories and groups sources of threonine contribution to the average Polish diet (food categories and groups contributed at least 0.2% of threonine), Table S8. Food categories and groups sources of tryptophan contribution to the average Polish diet (food categories and groups contributed at least 0.2% of tryptophan), Table S9. Food categories and groups sources of phenylalanine contribution to the average Polish diet (food categories and groups contributed at least 0.2% of phenylalanine), Table S10. Food categories and groups sources of methionine contribution to the average Polish diet (food categories and groups contributed at least 0.2% of methionine), Table S11. Food categories and groups sources of cysteine contribution to the average Polish diet (food categories and groups contributed at least 0.2% of cysteine), Table S12. Food categories and groups sources of tyrosine contribution to the average Polish diet (food categories and groups contributed at least 0.2% of tyrosine), Table S13. Food categories and groups sources of arginine contribution to the average Polish diet (food categories and groups contributed at least 0.2% of arginine), Table S14. Food categories and groups sources of glycine contribution to the average Polish diet (food categories and groups contributed at least 0.2% of glycine), Table S15. Food categories and groups sources of proline contribution to the average Polish diet (food categories and groups contributed at least 0.2% of proline), Table S16. Food categories and groups sources of aspartic acid contribution to the average Polish diet (food categories and groups contributed at least 0.2% of aspartic acid), Table S17. Food categories and groups sources of glutamic acid contribution to the average Polish diet (food categories and groups contributed at least 0.2% of glutamic acid), Table S18. Food categories and groups sources of serine contribution to the average Polish diet (food categories and groups contributed at least 0.2% of serine), Table S19. Food categories and groups sources of alanine contribution to the average Polish diet (food categories and groups contributed at least 0.2% of alanine).
