# Supplementary material for: Food Products as Sources of Protein and Amino Acids—The Case of Poland
Source: Nutrients. 2018 Dec 13;10(12):1977. doi: 10.3390/nu10121977 (PMC6315330; doi:10.3390/nu10121977)
Supplement: Supplementary file 1 [file nutrients-10-01977-s001.pdf]

Food Products as Sources of Protein and Amino Acids - the case of Poland

Supplementary Section

Table S1. Food group sources of protein contribution to the average Polish diet (food categories and groups contributed at least 0.2% of protein)
Table S2. Food categories and groups sources of leucine contribution to the average Polish diet (food categories and groups contributed at least 0.2% of leucine)
Table S3. Food categories and groups sources of isoleucine contribution to the average Polish diet (food categories and groups contributed at least 0.2% of isoleucine)
Table S4. Food categories and groups sources of valine contribution to the average Polish diet (food categories and groups contributed at least 0.2% of valine)
Table S5. Food categories and groups sources of lysine contribution to the average Polish diet (food categories and groups contributed at least 0.2% of lysine)
Table S6. Food categories and groups sources of histidine contribution to the average Polish diet (food categories and groups contributed at least 0.2% of histidine)
Table S7. Food categories and groups sources of threonine contribution to the average Polish diet (food categories and groups contributed at least 0.2% of threonine)
Table S8. Food categories and groups sources of tryptophan contribution to the average Polish diet (food categories and groups contributed at least 0.2% of tryptophan)
Table S9. Food categories and groups sources of phenylalanine contribution to the average Polish diet (food categories and groups contributed at least 0.2% of phenylalanine)
Table S10. Food categories and groups sources of methionine contribution to the average Polish diet (food categories and groups contributed at least 0.2% of methionine)
Table S11. Food categories and groups sources of cysteine contribution to the average Polish diet (food categories and groups contributed at least 0.2% of cysteine)
Table S12. Food categories and groups sources of tyrosine contribution to the average Polish diet (food categories and groups contributed at least 0.2% of tyrosine)
Table S13. Food categories and groups sources of arginine contribution to the average Polish diet (food categories and groups contributed at least 0.2% of arginine)
Table S14. Food categories and groups sources of glycine contribution to the average Polish diet (food categories and groups contributed at least 0.2% of glycine)
Table S15. Food categories and groups sources of proline contribution to the average Polish diet (' food categories and groups contributed at least 0.2% of proline)
Table S16. Food categories and groups sources of aspartic acid contribution to the average Polish diet (food categories and groups contributed at least 0.2% of aspartic acid)
Table S17. Food categories and groups sources of glutamic acid contribution to the average Polish diet (food categories and groups contributed at least 0.2% of glutamic acid)
Table S18. Food categories and groups sources of serine contribution to the average Polish diet (food categories and groups contributed at least 0.2% of serine)
Table S19. Food categories and groups sources of alanine contribution to the average Polish diet (food categories and groups contributed at least 0.2% of alanine)

Table S1. Food group sources of protein contribution to the average Polish diet

Food Group	Rank	% of Protein[1/] Contribution	Cumulative % of Protein Contribution
meat products	1	17.44	17.44
bread, rolls, bread products	2	16.49	33.93
red meat	3	9.91	43.84
poultry	4	9.71	53.55
cheese	5	5.64	59.19
milk	6	5.43	64.62
vegetables (excluding potatoes)	7	5.28	69.91
yoghurts and milk drinks	8	3.65	73.56
eggs	9	3.53	77.10
cottage cheese	10	3.34	80.43
flour, bran, cooking ingredients	11	3.10	83.53
potatoes	12	2.65	86.18
pizza, pasta, macaroni and other flour dishes	13	2.47	88.66
other meat	14	1.89	90.55
processed seafood	15	1.56	92.10
rice, cooked grains	16	1.09	93.20
fishes	17	0.99	94.18
fruits	18	0.93	95.11
chocolate	19	0.83	95.94
sweet bakery products	20	0.78	96.72
ready-to-eat cereal	21	0.77	97.50
desserts	22	0.53	98.03
sour cream	23	0.43	98.46
dried fruits an nuts	24	0.39	98.84
beer	25	0.32	99.17
juices	26	0.24	99.40
snacks	27	0.21	99.61

[1/] food categories and groups contributed at least 0.2% of protein

Table S2. Food categories and groups sources of leucine contribution to the average Polish diet

Food Categories<br>Food Groups	Rank	% of Leucine[1/]<br>Contribution	Cumulative % of Leucine<br>Contribution
**Food Categories**			
meat and meat products	1	39.85	39.85
grain products	2	22.10	61.95
milk and dairy products	3	20.02	81.97
vegetables	4	6.99	88.96
eggs	5	3.89	92.85
seafood	6	2.63	95.48
snacks and sweets	7	2.45	97.93
fruits	8	1.01	98.94
fats and oils	9	0.66	99.60
beverages, nonalcoholic	10	0.24	99.84
**Food Groups**			
meat products	1	18.39	18.39
bread, rolls, bread products	2	14.91	33.30
red meat	3	10.47	43.76
poultry	4	9.03	52.79
milk	5	6.15	58.95
cheese	6	5.96	64.91
vegetables (excluding potatoes)	7	4.84	69.75
yoghurts and milk drinks	8	4.24	73.98
eggs	9	3.89	77.87
cottage cheese	10	3.67	81.54
flour, bran, cooking ingredients	11	2.74	84.28
pizza, pasta, macaroni and other flour dishes	12	2.53	86.81
potatoes	13	2.16	88.97
other meat	14	1.96	90.93
processed seafood	15	1.56	92.49
rice, cooked grains	16	1.09	93.58
fishes	17	1.04	94.62
chocolate	18	0.86	95.47
ready-to-eat cereal	19	0.83	96.31
sweet bakery products	20	0.79	97.10
fruits	21	0.70	97.80
desserts	22	0.59	98.39
sour cream	23	0.49	98.88
dried fruits and nuts	24	0.31	99.19
snacks	25	0.21	99.41

[1/] food categories and groups contributed at least 0.2% of leucine

Table S3. Food categories and groups sources of isoleucine contribution to the average Polish diet

Food Categories<br>Food Groups	Rank	% of Isoleucine[1/]<br>Contribution	Cumulative % of<br>Isoleucine Contribution
**Food Categories**			
meat and meat products	1	41.32	41.32
grain products	2	21.31	62.63
milk and dairy products	3	19.05	81.68
vegetables	4	6.86	88.54
eggs	5	4.28	92.82
seafood	6	2.68	95.50
snacks and sweets	7	2.29	97.78
Fruits	8	1.01	98.79
fats and oils	9	0.70	99.49
nonalcoholic beverages	10	0.31	99.79
alcoholic beverages	11	0.20	99.99
**Food Groups**			
meat products	1	19.18	19.18
bread, rolls, bread products	2	14.82	34.00
red meat	3	10.29	44.28
poultry	4	10.06	54.34
milk	5	6.28	60.63
cheese	6	5.35	65.98
vegetables (excluding potatoes)	7	4.53	70.51
eggs	8	4.28	74.78
yoghurts and milk drinks	9	4.03	78.81
cottage cheese	10	3.39	82.20
pizza, pasta, macaroni and other flour dishes	11	2.43	84.63
flour, bran, cooking ingredients	12	2.37	87.00
potatoes	13	2.33	89.33
other meat	14	1.80	91.13
processed seafood	15	1.61	92.74
rice, cooked grains	16	1.05	93.79
fishes	17	1.04	94.84
chocolate	18	0.78	95.62
sweet bakery products	19	0.77	96.38
fruits	20	0.70	97.08
ready-to-eat cereal	21	0.64	97.72
desserts	22	0.55	98.27
sour cream	23	0.46	98.73
dried fruits and nuts	24	0.31	99.04
beer	25	0.20	99.24

[1/] food categories and groups contributed at least 0.2% of isoleucine

Table S4. Food categories and groups sources of valine contribution to the average Polish diet

Food Categories<br>Food Groups	Rank	% of Valine[1/]<br>Contribution	Cumulative % of<br>Valine Contribution
**Food Categories**			
meat and meat products	1	37.36	37.36
grain products	2	22.08	59.44
milk and dairy products	3	20.84	80.28
vegetables	4	7.76	88.05
eggs	5	4.32	92.37
seafood	6	2.72	95.08
snacks and sweets	7	2.50	97.58
fruits	8	1.10	98.69
fats and oils	9	0.69	99.38
nonalcoholic beverages	10	0.32	99.70
alcoholic beverages	11	0.30	99.99
**Food Groups**			
meat products	1	16.62	16.62
bread, rolls, bread products	2	15.30	31.92
poultry	3	9.47	41.39
red meat	4	9.45	50.84
milk	5	6.52	57.36
cheese	6	6.29	63.65
vegetables (excluding potatoes)	7	4.58	68.23
eggs	8	4.32	72.55
yoghurts and milk drinks	9	4.25	76.79
cottage cheese	10	3.79	80.58
potatoes	11	3.18	83.77
flour, bran, cooking ingredients	12	2.48	86.25
pizza, pasta, macaroni and other flour dishes	13	2.44	88.69
other meat	14	1.82	90.51
processed seafood	15	1.67	92.18
rice, cooked grains	16	1.14	93.32
fishes	17	1.02	94.34
chocolate	18	0.91	95.26
fruits	19	0.77	96.03
sweet bakery products	20	0.76	96.79
ready-to-eat cereal	21	0.72	97.51
desserts	22	0.62	98.13
sour cream	23	0.52	98.65
dried fruits and nuts	24	0.33	98.98
beer	25	0.30	99.27
snacks	26	0.21	99.48

[1/] food categories and groups contributed at least 0.2% of valine

Table S5. Food categories and groups sources of lysine contribution to the average Polish diet

Food Categories<br>Food Groups	Rank	% of Lysine[1/] Contribution	Cumulative % of Lysine Contribution
**Food Categories**			
meat and meat products	1	49.19	49.19
milk and dairy products	2	21.47	70.66
grain products	3	10.96	81.62
vegetables	4	7.53	89.15
eggs	5	3.32	92.47
seafood	6	3.19	95.67
snacks and sweets	7	2.14	97.80
fruits	8	0.97	98.77
fats and oils	9	0.71	99.48
nonalcoholic beverages	10	0.33	99.81
**Food Groups**			
meat products	1	22.30	22.30
poultry	2	12.41	34.72
red meat	3	12.30	47.01
bread, rolls, bread products	4	7.47	54.49
milk	5	7.42	61.91
cheese	6	6.44	68.35
vegetables (excluding potatoes)	7	4.96	73.31
yoghurts and milk drinks	8	4.28	77.59
cottage cheese	9	3.33	80.91
eggs	10	3.32	84.24
potatoes	11	2.57	86.81
other meat	12	2.17	88.98
processed seafood	13	1.89	90.87
pizza, pasta, macaroni and other flour dishes	14	1.44	92.31
fishes	15	1.28	93.59
flour, bran, cooking ingredients	16	1.13	94.72
chocolate	17	0.80	95.52
fruits	18	0.75	96.27
desserts	19	0.62	96.89
rice, cooked grains	20	0.61	97.50
sour cream	21	0.53	98.03
sweet bakery products	22	0.52	98.55
ready-to-eat cereal	23	0.30	98.85
dried fruits and nuts	24	0.22	99.07
juices	25	0.21	99.28
snacks	26	0.20	99.48

[1/] food categories and groups contributed at least 0.2% of lysine

Table S6. Food categories and groups sources of histidine contribution to the average Polish diet

Food Categories<br>Food Groups	Rank	% of Histidine[1/] Contribution	Cumulative % of Histidine Contribution
**Food Categories**			
meat and meat products	1	46.57	46.57
grain products	2	19.22	65.79
milk and dairy products	3	17.02	82.81
vegetables	4	6.50	89.31
seafood	5	3.22	92.53
eggs	6	2.81	95.34
snacks and sweets	7	1.91	97.26
fruits	8	1.53	98.79
fats and oils	9	0.50	99.29
nonalcoholic beverages	10	0.39	99.68
alcoholic beverages	11	0.31	99.99
**Food Groups**			
meat products	1	21.24	21.24
bread, rolls, bread products	2	13.06	34.30
red meat	3	11.83	46.13
poultry	4	11.59	57.71
cheese	5	5.76	63.48
milk	6	5.02	68.50
vegetables (excluding potatoes)	7	4.70	73.20
yoghurts and milk drinks	8	3.23	76.43
cottage cheese	9	3.00	79.43
eggs	10	2.81	82.24
flour, bran, cooking ingredients	11	2.49	84.73
processed seafood	12	2.33	87.06
pizza, pasta, macaroni and other flour dishes	13	2.19	89.25
other meat	14	1.91	91.17
potatoes	15	1.81	92.97
fruits	16	1.22	94.19
rice, cooked grains	17	0.89	95.08
fishes	18	0.89	95.97
chocolate	19	0.68	96.65
sweet bakery products	20	0.63	97.28
ready-to-eat cereal	21	0.58	97.87
desserts	22	0.46	98.33
sour cream	23	0.37	98.70
dried fruits and nuts	24	0.31	99.02
beer	25	0.31	99.33
juices	26	0.20	99.53

[1/] food categories and groups contributed at least 0.2% of histidine

Table S7. Food categories and groups sources of threonine contribution to the average Polish diet

Food Categories Food Groups	Rank	% of Threonine[1/] Contribution	Cumulative % of Threonine Contribution
**Food Categories**			
meat and meat products	1	44.70	44.70
grain products	2	18.48	63.18
milk and dairy products	3	17.55	80.73
vegetables	4	7.81	88.54
eggs	5	4.02	92.56
seafood	6	2.77	95.33
snacks and sweets	7	2.35	97.68
fruits	8	1.14	98.83
fats and oils	9	0.58	99.41
nonalcoholic beverages	10	0.34	99.74
alcoholic beverages	11	0.25	99.99
**Food Groups**			
meat products	1	20.57	20.57
bread, rolls, bread products	2	12.74	33.31
red meat	3	12.29	45.61
poultry	4	9.82	55.43
vegetables (excluding potatoes)	5	5.33	60.76
milk	6	5.19	65.95
cheese	7	4.94	70.89
eggs	8	4.02	74.91
yoghurts and milk drinks	9	3.81	78.72
cottage cheese	10	3.62	82.34
potatoes	11	2.47	84.81
flour, bran, cooking ingredients	12	2.13	86.94
other meat	13	2.01	88.96
pizza, pasta, macaroni and other flour dishes	14	2.01	90.97
processed seafood	15	1.63	92.60
fishes	16	1.11	93.71
rice, cooked grains	17	0.96	94.68
chocolate	18	0.86	95.54
fruits	19	0.84	96.37
sweet bakery products	20	0.72	97.10
ready-to-eat cereal	21	0.63	97.73
desserts	22	0.54	98.27
sour cream	23	0.43	98.70
dried fruits and nuts	24	0.31	99.00
beer	25	0.25	99.25
snacks	26	0.22	99.48

[1/] food categories and groups contributed at least 0.2% of threonine

Table S8. Food categories and groups sources of tryptophan contribution to the average Polish diet

Food Categories<br>Food Groups	Rank	% of Tryptophan[1/]<br>Contribution	Cumulative % of<br>Tryptophan Contribution
**Food Categories**			
meat and meat products	1	41.39	41.39
grain products	2	19.20	60.58
milk and dairy products	3	18.87	79.46
vegetables	4	9.05	88.50
eggs	5	4.19	92.69
seafood	6	2.42	95.11
snacks and sweets	7	2.31	97.43
fruits	8	1.17	98.60
fats and oils	9	0.60	99.20
alcoholic beverages	10	0.51	99.70
nonalcoholic beverages	11	0.29	99.99
**Food Groups**			
meat products	1	18.35	18.35
bread, rolls, bread products	2	12.91	31.27
red meat	3	10.63	41.90
poultry	4	10.55	52.45
cheese	5	6.24	58.68
vegetables (excluding potatoes)	6	5.40	64.08
milk	7	5.37	69.45
eggs	8	4.19	73.64
yoghurts and milk drinks	9	3.69	77.33
potatoes	10	3.65	80.98
cottage cheese	11	3.58	84.56
pizza, pasta, macaroni and other flour dishes	12	2.34	86.89
flour, bran, cooking ingredients	13	2.07	88.97
other meat	14	1.85	90.82
processed seafood	15	1.48	92.29
rice, cooked grains	16	1.19	93.48
fishes	17	0.92	94.40
chocolate	18	0.81	95.22
fruits	19	0.80	96.02
sweet bakery products	20	0.77	96.79
ready-to-eat cereal	21	0.69	97.47
desserts	22	0.54	98.01
beer	23	0.51	98.51
sour cream	24	0.44	98.96
dried fruits and nuts	25	0.37	99.33
snacks	26	0.20	99.53

[1/] food categories and groups contributed at least 0.2% of tryptophan

Table S9. Food categories and groups sources of phenylalanine contribution to the average Polish diet

Food Categories<br>Food Groups	Rank	% of Phenylalanine[1/] Contribution	Cumulative % of Phenylalanine Contribution
**Food Categories**			
meat and meat products	1	35.30	35.30
grain products	2	26.26	61.56
milk and dairy products	3	19.21	80.77
vegetables	4	7.88	88.65
eggs	5	4.39	93.04
snacks and sweets	6	2.50	95.55
seafood	7	2.23	97.77
fruits	8	1.11	98.88
fats and oils	9	0.59	99.47
nonalcoholic beverages	10	0.30	99.77
alcoholic beverages	11	0.22	99.99
**Food Groups**			
bread, rolls, bread products	1	18.04	18.04
meat products	2	16.40	34.44
red meat	3	9.19	43.62
poultry	4	7.86	51.49
cheese	5	6.23	57.71
milk	6	5.66	63.37
vegetables (excluding potatoes)	7	5.20	68.57
eggs	8	4.39	72.96
yoghurts and milk drinks	9	3.89	76.85
cottage cheese	10	3.43	80.29
flour, bran, cooking ingredients	11	3.38	83.67
pizza, pasta, macaroni and other flour dishes	12	2.93	86.60
potatoes	13	2.68	89.28
other meat	14	1.85	91.13
processed seafood	15	1.35	92.48
rice, cooked grains	16	1.15	93.64
chocolate	17	0.88	94.51
sweet bakery products	18	0.86	95.37
fishes	19	0.86	96.23
fruits	20	0.76	96.98
ready-to-eat cereal	21	0.75	97.74
desserts	22	0.55	98.29
sour cream	23	0.44	98.73
dried fruits and nuts	24	0.35	99.08
snacks	25	0.22	99.30
beer	26	0.22	99.52

[1/] food categories and groups contributed at least 0.2% of phenylalanine

Table S10. Food categories and groups sources of methionine contribution to the average Polish diet

Food Categories<br>Food Groups	Rank	% of Methionine[1/] Contribution	Cumulative % of Methionine Contribution
**Food Categories**			
meat and meat products	1	44.24	44.24
grain products	2	20.01	64.25
milk and dairy products	3	18.36	82.61
vegetables	4	5.07	87.68
eggs	5	4.93	92.62
seafood	6	3.21	95.83
snacks and sweets	7	2.16	97.99
fruits	8	0.88	98.87
fats and oils	9	0.59	99.46
nonalcoholic beverages	10	0.40	99.86
**Food Groups**			
meat products	1	19.93	19.93
bread, rolls, bread products	2	13.69	33.63
poultry	3	11.37	44.99
red meat	4	11.13	56.13
cheese	5	5.60	61.73
milk	6	5.58	67.31
eggs	7	4.93	72.24
yoghurts and milk drinks	8	3.76	76.01
cottage cheese	9	3.42	79.42
vegetables (excluding potatoes)	10	3.27	82.69
flour, bran, cooking ingredients	11	2.42	85.11
pizza, pasta, macaroni and other flour dishes	12	2.15	87.26
processed seafood	13	1.97	89.23
other meat	14	1.81	91.04
potatoes	15	1.80	92.84
fishes	16	1.22	94.06
rice, cooked grains	17	1.11	95.16
sweet bakery products	18	0.77	95.94
chocolate	19	0.71	96.64
fruits	20	0.65	97.29
ready-to-eat cereal	21	0.64	97.93
desserts	22	0.54	98.46
sour cream	23	0.44	98.90
dried fruits and nuts	24	0.23	99.14
other nonalcoholic beverages	25	0.23	99.37

[1/] food categories and groups contributed at least 0.2% of methionine

Table S11. Food categories and groups sources of cysteine contribution to the average Polish diet

Food Categories<br>Food Groups	Rank	% of Cysteine[1/]<br>Contribution	Cumulative % of Cysteine<br>Contribution
**Food Categories**			
grain products	1	41.74	41.74
meat and meat products	2	31.39	73.13
milk and dairy products	3	8.50	81.63
vegetables	4	6.36	87.99
eggs	5	5.10	93.09
snacks and sweets	6	2.38	95.47
seafood	7	1.81	97.27
fruits	8	1.22	98.49
alcoholic beverages	9	0.80	99.29
nonalcoholic beverages	10	0.36	99.65
**Food Groups**			
bread, rolls, bread products	1	29.02	29.02
meat products	2	14.58	43.60
red meat	3	8.11	51.71
poultry	4	7.11	58.82
flour, bran, cooking ingredients	5	6.34	65.15
eggs	6	5.10	70.25
vegetables (excluding potatoes)	7	4.67	74.92
pizza, pasta, macaroni and other flour dishes	8	3.46	78.38
milk	9	2.81	81.19
cheese	10	2.14	83.33
yoghurts and milk drinks	11	2.12	85.44
potatoes	12	1.69	87.13
rice, cooked grains	13	1.63	88.77
other meat	14	1.59	90.36
cottage cheese	15	1.44	91.80
ready-to-eat cereal	16	1.29	93.09
processed seafood	17	1.04	94.13
sweet bakery products	18	0.96	95.09
fruits	19	0.87	95.96
beer	20	0.80	96.76
chocolate	21	0.78	97.54
fishes	22	0.76	98.31
desserts	23	0.37	98.67
dried fruits and nuts	24	0.35	99.02
snacks	25	0.26	99.29
sour cream	26	0.26	99.54
juices	27	0.20	99.74

[1/] food categories and groups contributed at least 0.2% of cysteine

Table S12. Food categories and groups sources of tyrosine contribution to the average Polish diet

Food Categories<br>Food Groups	Rank	% of Tyrosine[1/]<br>Contribution	Cumulative % of Tyrosine<br>Contribution
**Food Categories**			
meat and meat products	1	38.30	38.30
milk and dairy products	2	23.61	61.91
grain products	3	20.58	82.50
vegetables	4	6.25	88.75
eggs	5	4.04	92.79
snacks and sweets	6	2.66	95.44
seafood	7	2.47	97.92
fruits	8	0.91	98.83
fats and oils	9	0.71	99.54
nonalcoholic beverages	10	0.28	99.81
**Food Groups**			
meat products	1	17.73	17.73
bread, rolls, bread products	2	14.48	32.21
red meat	3	10.01	42.22
poultry	4	8.81	51.03
cheese	5	8.33	59.35
milk	6	6.16	65.51
cottage cheese	7	4.64	70.15
vegetables (excluding potatoes)	8	4.51	74.67
yoghurts and milk drinks	9	4.49	79.15
eggs	10	4.04	83.20
pizza, pasta, macaroni and other flour dishes	11	2.22	85.41
flour, bran, cooking ingredients	12	1.98	87.39
other meat	13	1.75	89.15
potatoes	14	1.73	90.88
processed seafood	15	1.51	92.39
rice, cooked grains	16	1.17	93.56
chocolate	17	1.03	94.59
fishes	18	0.96	95.55
ready-to-eat cereal	19	0.73	96.28
sweet bakery products	20	0.72	97.00
desserts	21	0.66	97.65
fruits	22	0.62	98.27
sour cream	23	0.53	98.80
dried fruits and nuts	24	0.30	99.10
snacks	25	0.26	99.36

[1/] food categories and groups contributed at least 0.2% of tyrosine

Table S13. Food categories and groups sources of arginine contribution to the average Polish diet

Food Categories Food Groups	Rank	% of Arginine[1/] Contribution	Cumulative % of Arginine Contribution
**Food Categories**			
meat and meat products	1	46.12	46.12
grain products	2	20.99	67.11
milk and dairy products	3	11.34	78.45
vegetables	4	9.63	88.08
eggs	5	4.06	92.14
seafood	6	2.82	94.96
snacks and sweets	7	2.38	97.35
fruits	8	1.60	98.94
nonalcoholic beverages	9	0.47	99.41
fats and oils	10	0.35	99.76
alcoholic beverages	11	0.23	99.99
**Food Groups**			
meat products	1	20.88	20.88
bread, rolls, bread products	2	14.32	35.20
red meat	3	12.12	47.31
poultry	4	10.85	58.16
vegetables (excluding potatoes)	5	7.22	65.38
eggs	6	4.06	69.44
cheese	7	3.51	72.95
milk	8	3.40	76.35
flour, bran, cooking ingredients	9	2.43	78.78
potatoes	10	2.41	81.19
yoghurts and milk drinks	11	2.36	83.55
other meat	12	2.28	85.83
cottage cheese	13	2.07	87.90
pizza, pasta, macaroni and other flour dishes	14	1.92	89.82
processed seafood	15	1.75	91.57
rice, cooked grains	16	1.55	93.12
fishes	17	1.07	94.19
sweet bakery products	18	0.91	95.10
fruits	19	0.90	96.01
chocolate	20	0.89	96.90
ready-to-eat cereal	21	0.77	97.67
dried fruits and nuts	22	0.69	98.36
desserts	23	0.37	98.73
juices	24	0.29	99.02
sour cream	25	0.25	99.27
beer	26	0.23	99.50
snacks	27	0.21	99.71

[1/] food categories and groups contributed at least 0.2% of arginine

Table S14. Food categories and groups sources of glycine contribution to the average Polish diet

Food Categories<br>Food Groups	Rank	% of Glycine[1/] Contribution	Cumulative % of Glycine Contribution
**Food Categories**			
meat and meat products	1	52.46	52.46
grain products	2	21.05	73.52
milk and dairy products	3	7.84	81.36
vegetables	4	7.52	88.88
seafood	5	3.36	92.24
eggs	6	2.86	95.10
snacks and sweets	7	1.96	97.06
fruits	8	1.61	98.67
beverages, nonalcoholic	9	0.58	99.25
alcoholic beverages	10	0.45	99.70
fats and oils	11	0.29	99.99
**Food Groups**			
meat products	1	21.03	21.03
poultry	2	15.39	36.42
bread, rolls, bread products	3	14.38	50.80
red meat	4	12.84	63.65
vegetables (excluding potatoes)	5	5.44	69.08
other meat	6	3.20	72.28
eggs	7	2.86	75.15
flour, bran, cooking ingredients	8	2.62	77.77
cheese	9	2.41	80.18
milk	10	2.27	82.45
potatoes	11	2.08	84.53
pizza, pasta, macaroni and other flour dishes	12	2.07	86.60
processed seafood	13	2.03	88.63
yoghurts and milk drinks	14	1.70	90.33
cottage cheese	15	1.46	91.79
fishes	16	1.32	93.12
rice, cooked grains	17	1.20	94.31
fruits	18	1.15	95.46
ready-to-eat cereal	19	0.78	96.24
sweet bakery products	20	0.74	96.98
chocolate	21	0.71	97.69
dried fruits and nuts	22	0.46	98.15
beer	23	0.45	98.61
juices	24	0.42	99.02
desserts	25	0.28	99.30
snacks	26	0.23	99.53

[1/] food categories and groups contributed at least 0.2% of glycine

Table S15. Food categories and groups sources of proline contribution to the average Polish diet

Food Categories<br>Food Groups	Rank	% of Proline[1/] Contribution	Cumulative % of Proline Contribution
**Food Categories**			
grain products	1	34.11	34.11
meat and meat products	2	26.65	60.76
milk and dairy products	3	24.80	85.56
vegetables	4	5.43	90.99
snacks and sweets	5	2.45	93.44
eggs	6	1.97	95.41
seafood	7	1.79	97.19
fruits	8	0.88	98.07
alcoholic beverages	9	0.84	98.91
fats and oils	10	0.78	99.69
nonalcoholic beverages	11	0.30	99.99
**Food Groups**			
bread, rolls, bread products	1	23.49	23.49
meat products	2	11.13	34.63
cheese	3	8.18	42.81
poultry	4	7.27	50.08
milk	5	7.23	57.31
red meat	6	6.58	63.89
flour, bran, cooking ingredients	7	5.04	68.94
yoghurts and milk drinks	8	4.74	73.67
cottage cheese	9	4.65	78.32
pizza, pasta, macaroni and other flour dishes	10	3.88	82.20
vegetables (excluding potatoes)	11	3.80	86.00
eggs	12	1.97	87.97
other meat	13	1.67	89.64
potatoes	14	1.63	91.27
processed seafood	15	1.14	92.41
rice, cooked grains	16	0.95	93.36
beer	17	0.84	94.20
chocolate	18	0.84	95.03
sweet bakery products	19	0.82	95.85
ready-to-eat cereal	20	0.74	96.59
desserts	21	0.68	97.27
fishes	22	0.64	97.91
fruits	23	0.62	98.53
sour cream	24	0.58	99.11
dried fruits and nuts	25	0.26	99.37

[1/] food categories and groups contributed at least 0.2% of proline

Table S16. Food categories and groups sources of aspartic acid contribution to the average Polish diet

Food Categories<br>Food Groups	Rank	% of Aspartic Acid[1/] Contribution	Cumulative % of Aspartic Acid Contribution
**Food Categories**			
meat and meat products	1	41.69	41.69
grain products	2	16.68	58.37
milk and dairy products	3	14.29	72.66
vegetables	4	13.85	86.51
eggs	5	3.47	89.98
fruits	6	2.97	92.95
seafood	7	2.80	95.74
snacks and sweets	8	2.33	98.08
nonalcoholic beverages	9	1.13	99.21
fats and oils	10	0.48	99.69
alcoholic beverages	11	0.28	99.96
**Food Groups**			
meat products	1	18.52	18.52
bread, rolls, bread products	2	11.38	29.90
poultry	3	10.72	40.62
red meat	4	10.45	51.07
vegetables (excluding potatoes)	5	7.12	58.19
potatoes	6	6.73	64.92
milk	7	4.49	69.41
cheese	8	4.18	73.59
eggs	9	3.47	77.06
yoghurts and milk drinks	10	3.05	80.11
cottage cheese	11	2.57	82.68
fruits	12	2.40	85.08
other meat	13	2.00	87.08
pizza, pasta, macaroni and other flour dishes	14	2.00	89.08
processed seafood	15	1.67	90.75
flour, bran, cooking ingredients	16	1.58	92.33
fishes	17	1.12	93.45
rice, cooked grains	18	1.04	94.50
chocolate	19	0.85	95.35
ready-to-eat cereal	20	0.67	96.02
other nonalcoholic beverages	21	0.63	96.66
sweet bakery products	22	0.63	97.28
dried fruits and nuts	23	0.57	97.85
juices	24	0.50	98.35
desserts	25	0.45	98.80
snacks	26	0.40	99.20
sour cream	27	0.35	99.56
beer	28	0.27	99.83

[1/] food categories and groups contributed at least 0.2% of aspartic acid

Table S17. Food categories and groups sources of glutamic acid contribution to the average Polish diet

Food Categories<br>Food Groups	Rank	% of Glutamic Acid[1/] Contribution	Cumulative % of Glutamic Acid Contribution
**Food Categories**			
grain products	1	33.82	33.82
meat and meat products	2	30.90	64.72
milk and dairy products	3	18.95	83.67
vegetables	4	7.26	90.93
eggs	5	2.40	93.33
snacks and sweets	6	2.38	95.71
seafood	7	1.96	97.67
fruits	8	1.01	98.68
fats and oils	9	0.60	99.28
nonalcoholic beverages	10	0.38	99.66
alcoholic beverages	11	0.33	99.99
**Food Groups**			
bread, rolls, bread products	1	23.16	23.16
meat products	2	13.97	37.13
red meat	3	7.78	44.91
poultry	4	7.75	52.66
cheese	5	6.20	58.85
milk	6	5.31	64.16
vegetables (excluding potatoes)	7	5.15	69.31
flour, bran, cooking ingredients	8	4.99	74.30
yoghurts and milk drinks	9	3.76	78.07
pizza, pasta, macaroni and other flour dishes	10	3.75	81.82
cottage cheese	11	3.68	85.50
eggs	12	2.40	87.89
potatoes	13	2.11	90.00
other meat	14	1.41	91.41
processed seafood	15	1.21	92.61
rice, cooked grains	16	1.12	93.73
sweet bakery products	17	0.86	94.59
chocolate	18	0.81	95.40
ready-to-eat cereal	19	0.81	96.21
fishes	20	0.74	96.95
fruits	21	0.68	97.63
desserts	22	0.55	98.18
sour cream	23	0.45	98.63
dried fruits and nuts	24	0.33	98.97
beer	25	0.33	99.30
juices	26	0.22	99.52

[1/] food categories and groups contributed at least 0.2% of glutamic acid

Table S18. Food categories and groups sources of serine contribution to the average Polish diet

Food Categories<br>Food Groups	Rank	% of Serine[1/] Contribution	Cumulative % of Serine Contribution
**Food Categories**			
meat and meat products	1	33.63	33.63
grain products	2	24.04	57.67
milk and dairy products	3	21.07	78.73
vegetables	4	7.69	86.42
eggs	5	5.62	92.04
snacks and sweets	6	2.67	94.71
seafood	7	2.61	97.32
fruits	8	1.30	98.62
fats and oils	9	0.67	99.29
nonalcoholic beverages	10	0.43	99.72
alcoholic beverages	11	0.27	99.99
**Food Groups**			
bread, rolls, bread products	1	16.93	16.93
meat products	2	15.09	32.02
red meat	3	8.36	40.38
poultry	4	8.30	48.68
cheese	5	6.50	55.18
milk	6	6.35	61.53
eggs	7	5.62	67.14
vegetables (excluding potatoes)	8	5.36	72.51
cottage cheese	9	4.16	76.67
yoghurts and milk drinks	10	4.07	80.73
flour, bran, cooking ingredients	11	3.09	83.83
potatoes	12	2.33	86.15
pizza, pasta, macaroni and other flour dishes	13	2.11	88.27
other meat	14	1.88	90.14
processed seafood	15	1.59	91.73
rice, cooked grains	16	1.18	92.91
fishes	17	1.01	93.92
chocolate	18	1.00	94.92
fruits	19	0.95	95.87
sweet bakery products	20	0.86	96.73
ready-to-eat cereal	21	0.72	97.45
desserts	22	0.61	98.06
sour cream	23	0.49	98.55
dried fruits and nuts	24	0.36	98.90
beer	25	0.27	99.18
juices	26	0.23	99.40
snacks	27	0.20	99.61
other nonalcoholic beverages	28	0.20	99.81

[1/] food categories and groups contributed at least 0.2% of serine

Table S19. Food categories and groups sources of alanine contribution to the average Polish diet

Food Categories<br>Food Groups	Rank	% of Alanine[1/]<br>Contribution	Cumulative % of Alanine<br>Contribution
**Food Categories**			
meat and meat products	1	48.67	48.67
grain products	2	19.16	67.83
milk and dairy products	3	11.90	79.73
vegetables	4	7.82	87.55
Eggs	5	4.28	91.82
Seafood	6	3.46	95.29
snacks and sweets	7	1.97	97.26
Fruits	8	1.45	98.71
nonalcoholic beverages	9	0.48	99.19
alcoholic beverages	10	0.41	99.60
fats and oils	11	0.39	99.99
**Food Groups**			
meat products	1	21.23	21.23
bread, rolls, bread products	2	13.11	34.34
poultry	3	12.89	47.23
red meat	4	12.11	59.35
vegetables (excluding potatoes)	5	5.58	64.93
eggs	6	4.28	69.20
milk	7	3.84	73.04
cheese	8	3.42	76.45
yoghurts and milk drinks	9	2.46	78.91
other meat	10	2.43	81.34
potatoes	11	2.24	83.58
cottage cheese	12	2.19	85.77
flour, bran, cooking ingredients	13	2.10	87.88
processed seafood	14	2.10	89.97
pizza, pasta, macaroni and other flour dishes	15	2.02	92.00
fishes	16	1.36	93.36
rice, cooked grains	17	1.11	94.47
fruits	18	1.09	95.56
ready-to-eat cereal	19	0.81	96.37
chocolate	20	0.72	97.09
sweet bakery products	21	0.70	97.79
beer	22	0.41	98.20
desserts	23	0.38	98.58
dried fruits and nuts	24	0.36	98.93
juices	25	0.30	99.23
sour cream	26	0.28	99.51

[1/] food categories and groups contributed at least 0.2% of alanine
